# Evaluation of reciprocal F1 crosses of Fayoumi with two exotic chicken breeds 1: additive and non-additive effects on egg production traits

**DOI:** 10.1007/s11250-023-03735-9

**Published:** 2023-09-19

**Authors:** Fikrineh Negash, Solomon Abegaz, Yosef Tadesse, Temesgen Jembere, Wondmeneh Esatu, Tadelle Dessie

**Affiliations:** 1 Adami Tulu Agricultural Research Center, P. O. Box 35, Batu, Ethiopia; 2 School of Animal and Range Sciences, Haramaya University, P. O. Box 138, Dire Dawa, Ethiopia; 3 Ethiopian Institute of Agricultural Research, P. O. Box 2003, Addis Ababa, Ethiopia; 4 International Livestock Research Institute, P.O. Box 5689, Addis Ababa, Ethiopia

**Keywords:** Crossbreeding, Fayoumi, Maternal effect, Purebred effect, Reciprocal effect

## Abstract

The present study estimates additive and non-additive effects on egg production traits in genotypes generated through pure mating and reciprocal crossing of Fayoumi (FM) with Koekoek (KK) and White Leghorn (WL). Age at first egg (AFE) and body weight at first egg (BWAFE) were determined when the first bird in the pen laid its first egg, and egg weight at first egg (EWAFE) was the average weight of eggs laid consecutively during the first 10 days. Egg number (EN) and egg weight (EW) were recorded daily from AFE to 40 weeks of age. Egg mass (EM) was the product of EN and EW. EN of hens initially housed and hens alive during the experiment were used to calculate hen-housed egg production (HHEP) and hen-day egg production (HDEP), respectively. All the traits showed statistically significant differences among the genotypes. The results revealed the importance of additive and non-additive effects, where purebred effect (PE), general combining ability (GCA), maternal effect (ME), specific combining ability (SCA), and residual reciprocal effect (RRE) significantly affected most of the traits. The KK and WL had a higher PE, and GCA was highest in KK, with FM and WL showing a higher ME. The FM x WL had higher SCA and RRE. The KK x FM and FM x WL outperformed their main and reciprocal crosses, respectively, and purebred contemporaries. Therefore, a synthetic breeding program involving KK as a sire and FM, WL, FM x WL, and KK x FM as a dam would be feasible.

## Introduction

In Ethiopia, the poultry improvement program, which aimed to enhance egg and meat production from indigenous chickens, was started in the early 1950s (Tadelle et al., 2000). The program mainly involved the importation of genetically superior exotic breeds for use as either purebred or to be crossed with unselected indigenous ones. As part of this effort, imported chickens were distributed to rural smallholder farmers through on-farm research and public extension systems. Until the agricultural research system started evaluating the performances of those breeds (FAO, 2019), documented studies related to the adaptability of the genotypes to the local environment did not exist (Wondmeneh et al., 2014). The previous poultry development strategy, which aimed to increase the productivity of indigenous chickens, has historically had limited success, despite the paucity of empirical evidence to support it. Its failure is due to various factors, including poor adaptation of the breeds to a harsh production environment, their high susceptibility to disease challenges, and poor on-farm management practices (Tadelle et al., 2000). The manifestation of this failure is that the contribution of exotic chickens to the total poultry population and egg production (for instance, 9.11 and 9.38% in 2020, respectively; CSA, 2021) remains at less than 10% after more than half a century of the program’s inception.

In a situation where the environment is sub-optimal to allow expression of the potential of exotic breeds, crossing them with locally adapted chicken breeds may ensure the concert of the genotype and the environment. The strategy could improve overall production efficiency by exploiting the breeds’ complementarity for high genetic merit in different traits (Simm et al., 2021). Crossbreeding also exploits non-additive genetic variation due to heterosis or hybrid vigor. Heterosis has long been utilized in poultry breeding programs to produce progenies that exhibit higher performances than the average of their parental breeds (Williams et al., 2002).

Crossbreeding brings genetic progress to the population, but the change is not always permanent (Galukande et al., 2013). Furthermore, regularly producing crossbred animals would not be sustainable due to the high cost of obtaining and maintaining exotic genetic materials required in such a crossbreeding system (Leroy et al., 2016). Synthetic breed formation is an alternative to a regular crossing system to bring long-lasting genetic progress in the population (Galukande et al., 2013). It provides the advantage of maintaining only one locally adapted population with all desirable traits of the breeds involved instead of two or more purebred flocks required for regular crossbreeding (Munisi et al., 2015). Designing a synthetic breed formation program necessitates an evaluation of genetic variations (additive and non-additive) and a better understanding of the mode of genetic inheritance for different traits to identify genetic stocks that would have a good combining ability (Falconer and Mackay, 1996).

Evaluating the breeds for their combining ability and estimation of crossbreeding effects requires systematic crossbreeding designs (Jakubec et al., 1987). Diallel crossing is one of these methods used in testing populations. Although the complete diallel is most efficient in giving detailed information about crossbreeding effects with a small number of breeds, it is demanding and costly when the number of parents increases (Wolf and Knížetová, 1994). As a result, Wolf et al. (1991) recommended simpler experimental designs with fewer purebred and crossbred populations. They further noted that there are good reasons to split breeds used as parents into two sets, where only crosses between these sets are of interest.

Substantial variations have existed among exotic chicken breeds imported into the country since the 1950s and evaluated under intensive and village production systems. Recent studies that evaluated one or more of those breeds (e.g., Lemlem and Tesfay, 2010; Geleta et al., 2013; Tadesse et al., 2013; Geleta and Abdulkadir, 2018; Senbeta and Balcha, 2020) revealed variation in performance among Fayoumi, Koekoek, and White Leghorn breeds. For instance, those breeds annually lay 144 − 159, 187 − 213, and 173, respectively. Furthermore, they are recognized to have different genetic merits. Fayoumi is an indigenous dual-purpose breed of Egypt, and it is known for its good scavenging ability, resistance to some infectious (viral and bacterial) diseases, and adaptation to harsh production environments (Besbes, 2009; Bekele et al., 2010) like local chickens of Ethiopia. However, the empirical evidence indicates its lower production performance than Koekoek (a large dual-purpose breed) and White Leghorn (an egg-type breed).

Reciprocal crossing of Fayoumi with Koekoek and White Leghorn would be worthwhile in passing on high-production genes from the latter two breeds to Fayoumi, which already has genes responsible for survival under harsh rural conditions. The reciprocal and purebred populations can be evaluated when the differences in gene frequencies are supposed to exist between sire and dam breeds. For these crosses, crossbreeding parameters such as mean, heterosis, general and specific combining ability, purebred and maternal effects, and residual reciprocal effects can be estimated as in a complete diallel (Jakubec et al., 1987; Wolf et al., 1991). The current study hypothesizes that crossbreds may perform better than purebreds in egg production and quality traits. This article is the first of two series reports on the findings of an experiment conducted to investigate the additive and non-additive genetic effects on egg production and quality traits for genotypes generated through pure mating and reciprocal crossing of Fayoumi with Koekoek and White Leghorn.

## Materials and methods

### Study location

The experiment was carried out at the poultry research farm of Haramaya University, which is located at an elevation of 2010 m above sea level, 9°26′ N latitude and 42°03′ E longitude (Senbeta, 2017). The rainfall pattern of the area is bimodal, with mean annual precipitation ranging from 600 to 1260 mm while mean temperatures ranging between 9.74 and 24.05 °C (Burga et al., 2020; Adem, 2021).

### Mating plan and incubation

Crossbreds were generated by artificially inseminating hens of Koekoek (KK) and White Leghorn (WL) with semen collected from Fayoumi (FM) cocks, and inseminating the hens of the later breed with semen collected from cocks of KK and WL. Semen collection and insemination were conducted following procedures described in our previous experiment (Negash et al., 2023). Purebreds were produced by naturally mating hens of each breed with cocks of their type.

Eggs were collected daily in a group and labeled based on the sire and dam of the mating to avoid a mix-up of eggs during hatching and chicks after hatching. Incubation was performed under a standard procedure using an incubator with 37.5 °C temperature and 55% relative humidity. Candling was performed on day 18 to identify fertile eggs. Eggs that contained living embryos were transferred to hatching trays and placed in a hatchery. At hatch, chicks were tagged and placed in brooding pens.

### Management of birds

From hatch to 20 weeks, birds were reared as described previously (Negash et al., 2023). At 21 weeks, hens of each genotype were randomly distributed into three pens as replications under a completely randomized design (CRD). The total number of chickens for each genotype is indicated in Table 2. The birds were offered a measured quantity of feed and clean water ad libitum throughout the study period. An adjustment to the amount of feed offered was made every week based on the development stages of the birds (NRC, 1994). Nutrient compositions of diets fed to experimental chickens during brooding (from hatching to 8 weeks), growing (from 9 to 20 weeks), and egg production (from 20 to 40 weeks) periods are presented in Table 1. Vaccines against Newcastle (NCD; HB1 and Lasota), infectious bursal (Gumboro), and Fowl Pox diseases were administered to the birds at appropriate stages. Treatments were also applied to the birds using anti-coccidial and anti-helminthic medications.

**Table 1:** Nutrient composition of diets fed to experimental birds

Nutrient composition	Starter ration	Grower ration	Layer ration
ME, kcal/kg	2800.00	2800.00	2750.00
CP, %	20.00	16.00	16.50
CF, %	5.67	5.64	5.21
EE, %	4.23	4.31	4.19
Ca, %	0.99	1.00	3.68
Available P, %	0.69	0.60	0.80
Lysine, %	1.00	0.80	0.71
Methionine, %	0.45	0.32	0.30

*ME* metabolizable energy, *CP* crude protein, *CF* crude fiber, *EE* ether extract (crude fat), *Ca* calcium, *P* calcium

### Traits recorded

Age at first egg (AFE) was considered the sexual maturity of the birds when the first bird in the pen laid its first egg. Body weight at first egg (BWAFE) was taken as the weight of the hens in the group at AFE. Egg weight at first egg (EWAFE) was the average weight of the first ten eggs laid consecutively in each pen. Egg number (EN) and egg weight (EW) were recorded daily from the onset of laying to 40 weeks of age (i.e., part production record). Hen-housed egg production (HHEP) was estimated as the percentage of EN divided by the product of hens initially housed and the number of days that the birds were in lay, while hen-day egg production (HDEP) was calculated as the percentage of EN divided by the product of hens alive during the experiment (corrected for mortality) and the number of days that the hens were in lay. Egg mass (EM) was determined as the product of EN per hen and average EW.

### Statistical analysis

For all the traits, data were analyzed using the PROC MIXED model procedure of JMP (SAS Institute Inc., 2018) to determine the difference between the genotype (i.e., genetic group) with genotype as the main factor. The mean differences among the genotypes were separated using Tukey’s HSD test. The Box-Cox transformation method (Box and Cox, 1964) was applied to transform data for non-normally distributed variables.

For the traits that showed significant differences among the genotypes, a diallel model developed by Henderson (1948) and applied by Harvey (1960)—a model recommended to be suitable for the analysis of both full and partial diallel experiments (Model B; Jakubec et al., 1987)—was employed to estimate genetic effects. Before this analysis, percentages were transformed to arcsine square root values. The effect of each parental breed was assumed as fixed, and hence all effects in the model were considered fixed effects (Nath et al., 2007). The statistical model used was:

$${y}_{\mathrm{hijk}}=\mu +{a}_{\mathrm{h}}+{p}_{\mathrm{ii}}+{g}_{\mathrm{i}}+{g}_{\mathrm{j}}+ {m}_{\mathrm{j}}+{s}_{\mathrm{ij}}+{r}_{\mathrm{ij}}+{e}_{\mathrm{hijk}}$$

where, ${y}_{\mathrm{hijk}}$ is the *k*th observation on the progeny of a mating between *i*th sire group and *j*th dam group in *h*th type of breeding (purebred or crossbred); $\mu$ is the overall population mean; ${a}_{\mathrm{h}}$ is an effect common to all progenies of *h*th type of breeding (purebred or crossbred); ${p}_{\mathrm{ii}}$ is the purebred effect (PE) common to all progenies of mating between *i*th sire group and *j*th dam group; ${g}_{\mathrm{i}}$ (${g}_{\mathrm{j}}$) is the general combining ability (GCA) for the *i*th (*j*th) breed; ${m}_{\mathrm{j}}$ is the maternal effect (ME) of *j*th dam breed; ${s}_{\mathrm{ij}}$ is the specific combining ability (SCA) in the progeny of *i*th and *j*th breed; ${r}_{\mathrm{ij}}$ is the residual reciprocal effect (RRE) in the progeny of *i*th sire group and *j*th dam group; and ${e}_{\mathrm{hijk}}$ is the random error term.

## Results

### Relative performance

Table 2 presents the relative performances of the purebreds and crossbreds for AFE, BWAFE, and EWAFE, where all the traits significantly differed among the genotypes. The FM x KK and WL started laying earlier and later than others, respectively, while the remaining genotypes had intermediate AFE. The FM x KK matured sexually earlier than its purebred contemporaries. At the beginning of laying, KK (1374 g) and KK x FM (1314 g) had the highest BW, followed by FM x KK (1206 g), WL x FM (1086 g), FM x WL (1060 g), WL (1050 g), and FM (1019 g). Reciprocal crosses of FM with KK showed higher BWAFE than FM, not KK. When the genotypes started laying, KK (38.59 g) and WL (36.86 g) laid the heaviest eggs. Crosses of these breeds as male parents with FM laid intermediate-sized eggs, while FM and its crosses with WL and KK as a male laid the lightest eggs. The KK x FM had a higher EWAFE than FM, not KK.

**Table 2:** Mean performance of the seven genotypes for age at first egg (AFE), body weight at first egg (BWAFE), and egg weight at first egg (EWAFE)

Genotype	HIH	HAE	AFE, days	BWAFE, g	EWAFE, g
Purebreds					
FM	23	22	164.67^ab^	1018.55^c^	33.99^bc^
KK	11	11	166.50^ab^	1373.81^a^	38.59^a^
WL	18	17	169.33^a^	1049.62^c^	36.86^ab^
Main F1 crosses					
FM♂ x KK♀	21	20	151.67^b^	1206.15^ab^	32.81^c^
FM♂ x WL♀	30	28	160.00^ab^	1060.23^c^	34.13^bc^
Reciprocal F1 crosses					
KK♂ x FM♀	16	15	160.00^ab^	1314.10^a^	35.04^abc^
WL♂ x FM♀	30	30	167.00^ab^	1086.05^bc^	35.37^abc^
SEM			9.20	91.75	2.26
* P* value			0.0406	< 0.0001	0.0002

Means not connected by the same superscript in a column are significantly different at *P* < 0.05; *SEM* pooled standard error of the mean, *FM* Fayoumi, *KK* Koekoek, *WL* White Leghorn, *HIH* number of hens initially housed, *HAE* number of hens alive to the end of the experiment

The relative performances of the genotypes were also assessed for EN, EW, HHEP, HDEP, and EM (Table 3). The traits showed statistically significant differences among genotypes (*P* < 0.001). The KK x FM had the highest EN (both hen-housed and hen-day basis), followed by WL and FM x WL, with the other genotypes having a lower EN. The crossbred with the highest EN outperformed its purebred contemporaries and FM x KK. For eggs collected throughout the study period, WL and KK had the heaviest eggs, while main and reciprocal crosses of FM with KK and WL laid the medium-sized eggs (43 − 44 g), with FM laying the lightest eggs (40 g). The WL and KK x FM had the highest egg production rate (HHEP and HDEP), followed by FM x WL, KK, FM, and WL x FM, but FM x KK performed the least. The KK x FM and WL had the highest EM (estimated on both hen-housed and hen-day basis), while KK, FM x WL, and FM x KK showed intermediate performance, with WL x FM and FM having the lowest EM. The KK x FM showed a higher egg production rate and EM than its purebred contemporaries and FM x KK.

**Table 3:** Mean performance of the seven genotypes for egg weight (EW), total egg number (EN), hen-housed egg production (HHEP), hen-day egg production (HDEP), and egg mass (EM)

Genotype	EW, g	Total EN per hen, n		Egg production rate, %		EM, g per hen	
		Hen-housed	Hen-day	HHEP	HDEP	Hen-housed	Hen-day
Purebreds							
FM	40.30^d^	43.5^ cd^	45.8^ cd^	37.58^ cd^	39.68^b^	1816.89^e^	1916.99^e^
KK	45.93^a^	44.6^bcd^	44.6^ cd^	38.88^bc^	38.91^b^	2193.10^abcd^	2208.64^bcd^
WL	46.18^a^	50.5^ab^	51.8^ab^	45.21^a^	46.36^a^	2404.61^ab^	2467.08^ab^
Main F1 crosses							
FM♂ x KK♀	44.19^b^	43.7^ cd^	43.9^d^	33.81^d^	33.92^c^	2010.43^cde^	2017.09^cde^
FM♂ x WL♀	43.71^bc^	48.5^abc^	49.5^bc^	40.03^bc^	40.82^b^	2179.56^bcd^	2223.89^bc^
Reciprocal F1 crosses							
KK♂ x FM♀	44.49^b^	52.8^a^	55.5^a^	43.66^ab^	45.89^a^	2445.03^a^	2583.75^a^
WL♂ x FM♀	43.24^c^	42.0^d^	42.9^d^	37.57^ cd^	37.57^bc^	1931.29^de^	1931.56^de^
SEM	0.52	3.0	3.0	2.50	2.56	134.69	138.16
*P* value	< 0.0001	< 0.0001	< 0.0001	< 0.0001	< 0.0001	< 0.0001	< 0.0001

Means not connected by the same superscript in a column are significantly different at *P* < 0.05; *SEM* pooled standard error of the mean, *FM* Fayoumi, *KK* Koekoek, *WL* White Leghorn

**Fig. 1:**
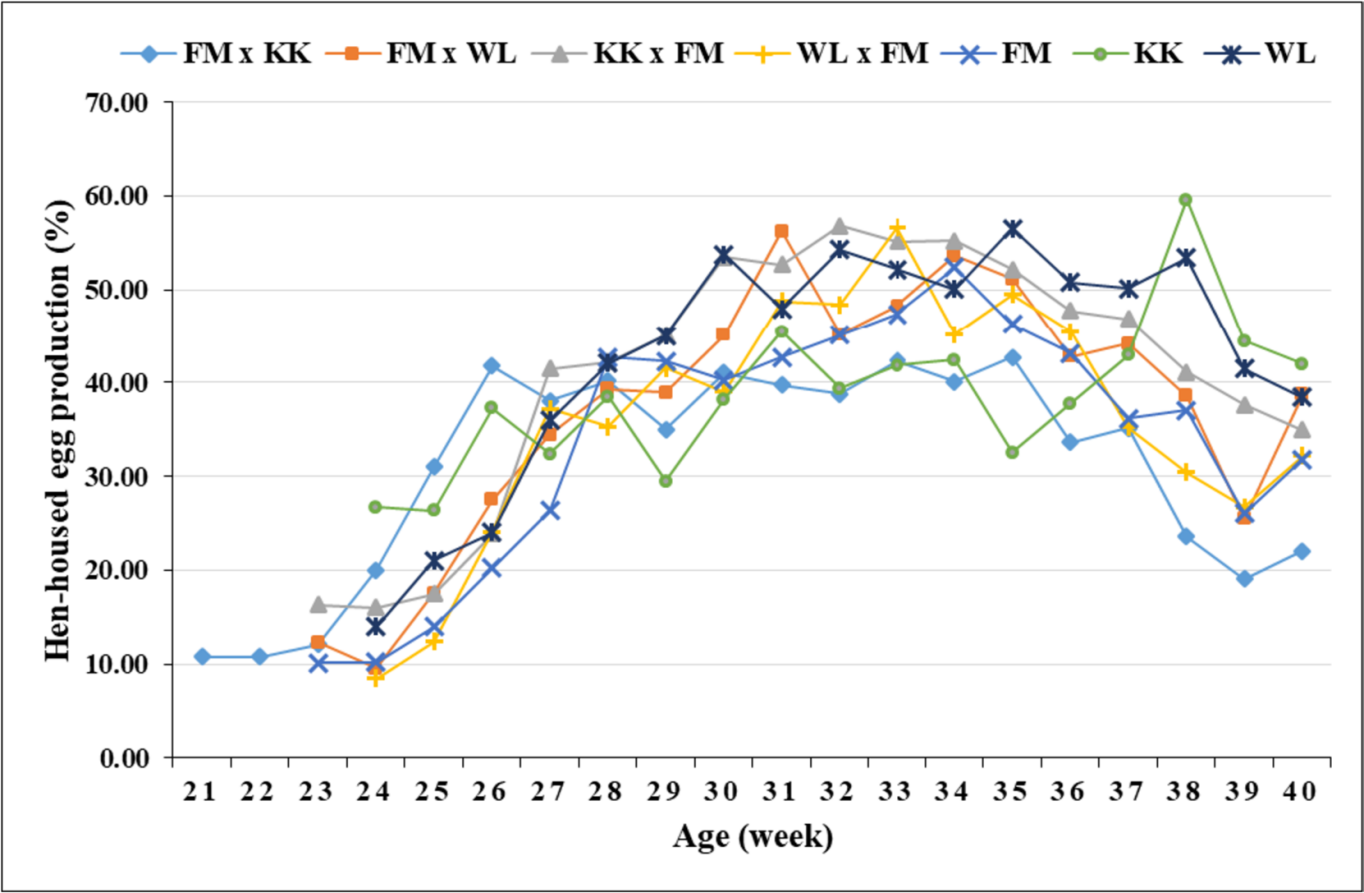
Hen-housed egg production (%) of the genotypes over time

**Fig. 2:**
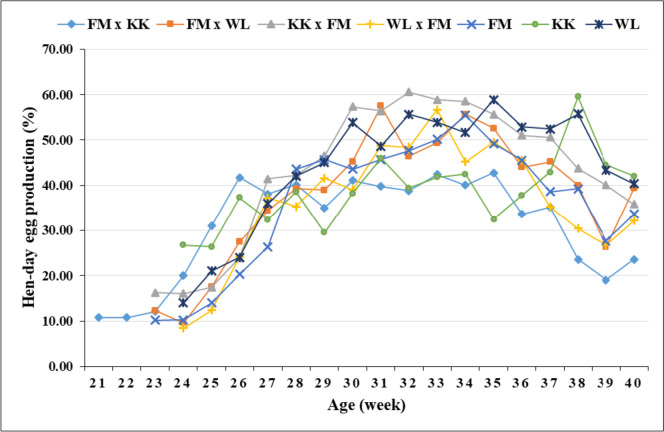
Hen-day egg production (%) of the genotypes over time

**Fig. 3:**
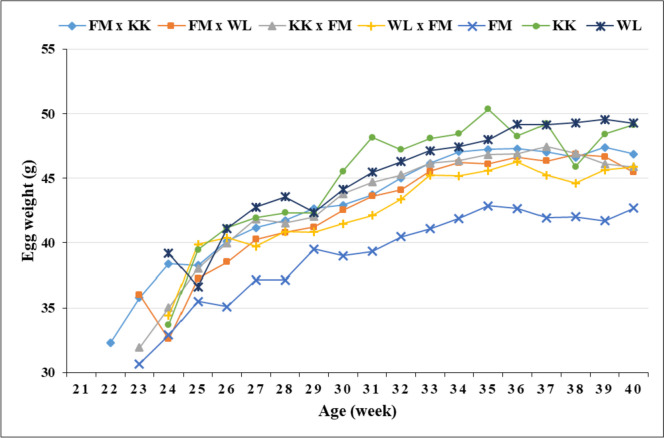
Egg weight of the genotypes over time

Peak egg production rate was attained by most genotypes at 31 − 35 weeks of age, with KK reaching the peak later at week 38 (Figs. 1 and 2). Most genotypes attained a higher egg production rate (> 50%) at 27 − 29 weeks, with FM x KK and KK achieving the same rate earlier (week 26) and later (week 32), respectively. In most genotypes, EW showed an increasing trend with layers’ age (Fig. 3).

### Additive genetic variance

#### Purebred effect

The PE significantly affected all egg production traits (Table 4). For EWAFE and BWAFE, KK showed positive and the highest PE, followed by WL and FM. Statistically comparable PE was found in KK and WL for EW and EM, with FM having lower and negative values. On the other hand, WL showed a higher PE in EN, HHEP, and HDEP, while KK and FM had lower and negative estimates.

**Table 4:** Mean estimates of different genetic effects for egg production traits

Genetic effect	BWAFE	EWAFE	EN	EW	EM	HHEP	HDEP
Overall mean, *μ*	1139.55 ± 15.77	35.41 ± 0.30	46.7 ± 0.4	43.86 ± 0.09	2144.45 ± 19.74	0.675 ± 0.004	0.685 ± 0.004
Heterosis,$\overline{{\varvec{h}} }$							
${{\varvec{a}}}_{1}$	− 18.65 ± 15.77	1.07 ± 0.30	− 0.4 ± 0.4	− 0.05 ± 0.09	− 0.27 ± 19.74	0.012 ± 0.004	0.014 ± 0.004
${{\varvec{a}}}_{2}$	18.65 ± 15.77	− 1.07 ± 0.30	0.4 ± 0.4	0.05 ± 0.09	0.27 ± 19.74	− 0.012 ± 0.004	− 0.014 ± 0.004
PE	***	**	***	***	***	***	***
${{\varvec{p}}}_{11}$	− 101.67 ± 39.78^b^	− 2.50 ± 0.86^b^	− 2.7 ± 1.3^b^	− 3.50 ± 0.22^b^	− 318.10 ± 60.74^b^	− 0.035 ± 0.012^b^	− 0.024 ± 0.013^b^
${{\varvec{p}}}_{22}$	254.66 ± 48.72^a^	2.09 ± 0.86^a^	− 1.7 ± 1.4^b^	2.19 ± 0.29^a^	108.85 ± 82.54^a^	− 0.017 ± 0.013^b^	− 0.029 ± 0.014^b^
${{\varvec{p}}}_{33}$	− 71.51 ± 40.76^b^	0.40 ± 0.86^ab^	4.3 ± 1.3^a^	2.38 ± 0.22^a^	267.14 ± 61.67^a^	0.051 ± 0.012^a^	0.051 ± 0.013^a^
GCA	***	NS	***	***	***	*	***
${{\varvec{g}}}_{1}$	− 50.87 ± 30.33^b^	− 0.58 ± 0.80^a^	− 0.7 ± 1.1^b^	0.03 ± 0.20^b^	− 32.37 ± 51.58^b^	− 0.015 ± 0.010^b^	− 0.017 ± 0.010^b^
${{\varvec{g}}}_{2}$	241.84 ± 54.82^a^	− 0.05 ± 1.13^a^	5.4 ± 1.6^a^	0.95 ± 0.29^a^	309.53 ± 74.16^a^	0.036 ± 0.015^a^	0.051 ± 0.015^a^
${{\varvec{g}}}_{3}$	− 162.09 ± 39.43^b^	1.22 ± 1.13^a^	− 4.2 ± 1.6^b^	− 0.99 ± 0.29^c^	− 247.89 ± 73.69^c^	− 0.008 ± 0.015^ab^	− 0.019 ± 0.015^b^
ME	**	***	***	*	***	***	***
${{\varvec{m}}}_{1}$	31.08 ± 16.92^a^	1.17 ± 0.39^a^	1.2 ± 0.6^b^	− 0.07 ± 0.10^ab^	59.48 ± 26.51^b^	0.028 ± 0.005^a^	0.032 ± 0.005^a^
${{\varvec{m}}}_{2}$	− 71.90 ± 25.75^b^	− 1.50 ± 0.55^b^	− 6.1 ± 0.8^c^	− 0.19 ± 0.14^b^	− 286.33 ± 36.51^c^	− 0.072 ± 0.007^b^	− 0.086 ± 0.007^b^
${{\varvec{m}}}_{3}$	− 16.92 ± 20.49^ab^	− 0.83 ± 0.55^b^	3.7 ± 0.8^a^	0.31 ± 0.14^a^	168.31 ± 37.49^a^	0.018 ± 0.007^a^	0.024 ± 0.008^a^
SCA	***	NS	**	***	***	NS	NS
${{\varvec{s}}}_{12}$	− 67.61 ± 16.58^b^	0.40 ± 0.43^a^	− 1.1 ± 0.6^b^	− 0.43 ± 0.11^b^	− 80.55 ± 27.29^b^	0.001 ± 0.005^a^	− 0.003 ± 0.005^a^
${{\varvec{s}}}_{13}$	120.61 ± 13.19^a^	− 0.40 ± 0.43^a^	1.2 ± 0.6^a^	0.43 ± 0.11^a^	84.49 ± 28.02^a^	0.000 ± 0.005^a^	0.004 ± 0.005^a^
RRE	NS	NS	**	NS	**	**	***
${{\varvec{r}}}_{12}$	− 2.44 ± 16.58^a^	0.21 ± 0.43^a^	− 0.9 ± 0.6^b^	− 0.08 ± 0.11^a^	− 41.84 ± 27.29^b^	− 0.004 ± 0.005^b^	− 0.005 ± 0.005^b^
${{\varvec{r}}}_{13}$	11.31 ± 13.19^a^	0.38 ± 0.43^a^	1.6 ± 0.6^a^	0.04 ± 0.11^a^	71.82 ± 28.02^a^	0.018 ± 0.005^a^	0.022 ± 0.005^a^

Estimates not connected by the same superscript in a column are significantly different at *P* < 0.05; *NS* not significant, *P < 0.05, **P < 0.01, ***P < 0.001; the subscripts 1, 2, and 3 indicate FM, KK, and WL purebreds, respectively; the subscripts 12 and 13 represent FM x KK and FM x WL crossbreds, respectively

#### General combining ability

Variation due to GCA was significant in all traits except EWAFE (Table 4), where KK exhibited positive and the highest GCA, while the other two breeds had negative and lower values. The FM and WL showed comparably negative values in BWAFE, EN, HHEP, and HDEP. Compared with KK, these breeds also had intermediate and lower GCA in EW and EM, respectively.

#### Maternal effect

The ME was significant in all egg production traits (Table 4). The FM had positive and the highest ME for BWAFE and EWAFE, while WL showed higher values for EN and EW. Compared with KK, for which ME was negative, the values were higher and positive in both FM and WL for HHEP and HDEP.

### Non-additive genetic variance

#### Heterosis

The overall heterosis, the difference between ${a}_{2}$ and ${a}_{1}$ (Table 4), was positive in BWAFE, EN, EW, and EM. On the other hand, EWAFE, HHEP, and HDEP had negative heterosis.

#### Specific combining ability

The SCA effect was highly significant (*P* < 0.01) in BWAFE, EN, EW, and EM but non-significant in the other traits (Table 4). Compared with FM x KK, positive and a higher SCA was recorded in FM x WL.

#### Residual reciprocal effect

Variation due to RRE was highly significant (*P* < 0.01) in EN, EM, HHEP, and HDEP (Table 4). For these traits, the estimates were higher and positive in FM x WL, but FM x KK exhibited lower and negative RRE values.

## Discussion

### Relative performance

Egg production is a complex metric trait that exhibits several variations during the layer’s production cycle (Khawaja et al., 2013). Several egg production traits can define the production performance of the birds. In the current study, all studied egg production traits significantly differed among the genotypes. Contrary to Khawaja et al. (2013), who reported earlier sexual maturity in Fayoumi than in Rhode Island Red (RIR) and their reciprocal crosses, AFE was shorter in FM x KK than in FM and KK. However, like the current results, crossbred chickens (Williams et al., 2002; Lalev et al., 2014; Amao, 2017; Balcha et al., 2021) and ducks (Padhi, 2010) showed shorter AFE than their purebred contemporaries, indicating that crossbreeding improves sexual maturity. Reciprocal crosses of Dominant Black with Fulani ecotype of Nigeria started laying earlier than Fulani but not Dominant Black (Sola-Ojo and Ayorinde, 2011).

The large dual-purpose breed and reciprocal crosses involving it (i.e., FM x KK and KK x FM) attained higher BWAFE than other genotypes. These genotypes also showed better growth performance than FM, WL, and their reciprocal crosses before the laying stage (Negash et al., 2023). In an experiment where Dominant Red Barred was crossed reciprocally with an improved Ethiopian Horro ecotype, the results showed a higher BWAFE performance in the former, followed by the reciprocal crosses, with the Horro ecotype performing the least (Hussen et al., 2020). Like this report, reciprocal crosses of FM with KK had a higher BWAFE than FM, but not KK, suggesting that KK passed on genes responsible for growth performance to the crossbred progenies better than FM. In the study involving reciprocal mating of Horro with KK and Kuroiler (Taye et al., 2022), reciprocal crosses of Kuroiler and Horro had the highest BWAFE, followed by Kuroiler and reciprocal crosses of KK with Horro, but purebred KK and Horro showed the lowest performance. However, Sola-Ojo and Ayorinde (2011) reported that reciprocal crosses outweighed both purebreds when the birds started laying. Reciprocal crosses of FM and WL also showed higher BWAFE than the two purebreds (Balcha et al., 2021).

At the start of laying and throughout the study period, KK and WL laid the heaviest eggs, with FM having the lightest eggs. Balcha et al. (2021) also reported that WL and FM had the highest and the lowest EWAFE, respectively, with the reciprocal crosses involving them showing intermediate performance. In the diallel crosses involving FM, Sinai, WL, and RIR (Saadey et al., 2008), WL and FM had the heaviest and lightest eggs, respectively. Relatively better EWAFE was obtained in crosses involving males of KK and WL with FM females, compared with their main cross counterparts, which indicates the effect of sex-linked genes. Reciprocal crosses of FM with KK and WL also had a higher EW than FM but not KK and WL, respectively, which suggests that KK and WL transmitted their higher genes for the trait to the crossbreds than FM. On the contrary, reciprocal crosses laid heavier eggs than the purebreds (Sola-Ojo and Ayorinde, 2011). The current results showed similar trends to Soliman et al. (2020), who reported the heaviest, medium, and lowest EW in Lohmann White, reciprocal crosses of Alexandria and Lohmann White, and Alexandria, respectively.

Intensive selection for egg production in laying strains has resulted in birds with low BW but higher egg production potential (Thiruvenkadan et al., 2010), which has often supported by the present results, where WL showed the lower BWAFE (Table 2) and the higher EW, EN, HHEP, and HDEP (Table 3). Besides, WL had lower growth performance during the brooding and growing stages (Negash et al., 2023). The WL outperformed the crossbreds, which might suggest that the additive effects would be more significant than the non-additive effects.

The KK x FM outperformed its purebred contemporaries and FM x KK in EN, HHEP, HDEP, and EM, indicating a higher combining ability of the two purebreds in the reciprocal than the main crosses. In crosses involving KK and FM, it would be advantageous to use them as a sire and dam, respectively, to exploit heterosis. In Taye et al. (2022), reciprocal crosses of Horro and Kuroiler showed the highest performances in EN, HHEP, and HDEP, while Horro performed the least with KK, Kuroiler, and reciprocal crosses between KK and Horro exhibiting intermediate performance. The crosses between indigenous and non-indigenous ducks mostly outperformed their purebred parents in EW and duck-day egg production (Padhi, 2010; Padhi et al., 2023). In the current study, FM x WL performed better than FM, WL, and WL x FM in EN, HHEP, and EM. The crosses of FM males and WL females would benefit from heterosis in egg production. The higher performances of KK x FM and FM x WL over FM x KK and WL x FM, respectively, in different traits might suggest the existence of sex-linked effects. Crosses of indigenous and exotic breeds mostly outperformed both purebreds, while their reciprocals performed better than the indigenous breed but not exotic ones (Amao, 2017; Hussen et al., 2020). In some studies (Khalil et al., 2004; Soliman et al., 2020; El-Tahawy and Habashy, 2021), reciprocal crosses of exotic and indigenous breeds performed better than indigenous breeds but not exotic ones for different traits. In Wolde et al. (2021), crosses between exotic SS-RIR and local chicken of Ethiopia mostly outperformed local chicken but not SS-RIR.

The age at which the genotypes attained peak egg production in the present study is in close agreement with the period for peak production (31 − 34 weeks; Ünver et al., 2004). However, Wolde et al. (2021) reported 27 weeks for crossbred and exotic chickens and 47 weeks for local chickens. Usually, the egg production curve for layers increases rapidly during the first 8 or 9 weeks of production and decreases slowly after a certain period of maintaining constant production (Thiruvenkadan et al., 2010). The genotype (i.e., KK x FM) with flatter egg production from 30 to 35 weeks of age (Figs. 1 and 2) also showed the highest production (EN, HHEP, HDEP, and EM; Table 3), with the other genotypes having fluctuating curves. In good agreement with the present results, EW showed an increasing trend with layers’ age in different genotypes of ducks (Padhi, 2010; Padhi et al., 2023) and chickens (Ni et al., 2022).

### Additive genetic variance

A better understanding of additive and non-additive genetic variances and their mode of inheritance is crucial for designing appropriate synthetic breeding or any crossbreeding program. The PE, GCA, and ME are effects of additive genetic variance. In the present study, these variances significantly affected all egg production traits except EWAFE, where the GCA effect was non-significant. The higher PE in KK and WL for most traits would manifest higher additive and dominance genetic variances and the existence of favorable alleles in these breeds, according to Nath et al. (2014). The results also suggest that these breeds might have undergone improvements in egg production. Furthermore, the higher PE implies that these breeds may have higher GCA, ME, or both variances (Nath et al., 2007), which is confirmed partly by the present results.

The GCA is defined as the average performance of a breed if the breed is combined with others in a cross (Falconer and Mackay, 1996). Significant variation due to GCA could suggest the importance of additive effects of all parental gametes (i.e., equivalent to the breeding value of a parent; Nath et al., 2007). Like the current study, Padhi (2010) reported significant GCA in AFE, EW, and duck-day egg production. Positive and higher GCA in KK for most traits indicates the accumulation of favorable alleles for egg production in KK relative to FM and WL, which had negative and lower values. Desirable additive genes available in this breed would likely pass on to progenies having this breed as a parent (i.e., good combining ability). Higher GCA also demonstrates that the purebred mean is superior to the general mean and has fewer environmental effects, whereas lower GCA indicates that the purebred mean does not differ more from the mean of the crosses (Fasahat et al., 2016). Contrary to the current results, Saadey et al. (2008) reported positive GCA for EW, HHEP, and EN in Sinai, FM, WL, and RIR purebreds. For AFE, egg production, and EW, WL gave the highest (positive) GCA compared with local Iraqi Brown, which exhibited the lowest (negative) value (Razuki and AL-Shaheen, 2011).

The ME, as opposed to the direct additive genetic contribution of the mother, is the effect of genes of the dam on its offspring’s performance through the environment that the mother provides (Lotfi et al., 2012). This effect on the progeny’s performance can be a function of maternal variability brought on by genetic, environmental, or a combination of the two factors (Grosso et al., 2010). The ME measures a breed’s ability for pre-natal and post-natal mothering (Henderson, 1948; Harvey, 1960). As any ME on artificially incubated chicks must be the residual effect of the hen reflected in egg characteristics at laying, this effect in birds is distinct from that in mammals (Lotfi et al., 2012). Factors that may determine ME are egg size, incubation environment, egg composition, maternal antibodies, and cytoplasmic or mitochondrial inheritance (Barbato and Vasilatos, 1991).

According to different authors (Nath et al., 2007, 2014; Rajkumar et al., 2011), the significant variation in ME suggests the influence of maternal additive and dominance gene effects. Other authors (El-Tahawy, 2020; Hussen et al., 2020) also reported a significant variation due to ME in EM and EN. The higher ME observed in FM and WL for different traits would suggest that using these breeds as a dam in a crossbreeding program aiming to improve egg production traits for which the breeds showed a higher ME is appropriate.

### Non-additive genetic variance

Heterosis and SCA are the main effects of non-additive gene action. Variation due to RRE also indicates non-additive genetic variation resulting from an interaction between sex and autosomal chromosomes (Wearden et al., 1967). The overall heterosis, the difference between the overall mean for crossbreds and the overall mean for purebreds (Jakubec et al., 1987; Onofri et al., 2021), was found to be positive in BWAFE, EN, EW, and EM, which mean that crossbreds outperformed purebreds in those traits. For the other traits that showed negative heterosis, on the other hand, the purebreds would perform better than the crossbreds.

The SCA measures the average inferiority or superiority of particular crosses relative to the average performance of the purebreds involved in the cross (Henderson, 1948; Harvey, 1960; Falconer and Mackay, 1996). In close agreement with the present findings, where SCA significantly affected some traits, Padhi (2010) noted that SCA affected AFE, EW, and duck-day egg production. Fairfull et al. (1983) also found significant SCA effects for AFE, HHEP, HDEP, and EW. Variation in SCA is due to the non-additive effect of putting gametes together in pairs to make the F1 crosses, which involves either dominance or epistatic interaction or a combination of the two (Eisen et al., 1967; Rajkumar et al., 2011; Onofri et al., 2021). Significant SCA variation suggests the likelihood of improving the traits by utilizing management practices (Musa et al., 2015). Positive and higher SCA in FM x WL for BWAFE, EN, EW, and EM means the appropriateness of using this genotype as a dam in any crossbreeding programs. Saadey et al. (2008) reported that SCA was a significant source of variation for EW, HHEP, and EN, where FM x Sinai and WL x RIR had positive values.

Reciprocal effects reflect variations in gene frequencies between sire and dam breeds in the presence of additive maternal and/or dominant maternal effects (Eisen et al., 1983). These effects are general and specific (residual) reciprocal effects. Henderson (1948) interpreted the former as an effect due to maternal effects and the latter as a variance due to sex-linked effects. On the other hand, Eisen et al. (1967) and Eisen et al. (1983) interpreted general reciprocal effect as a variance containing additive sex-linked and maternal effects and viewed RRE as a variance resulting from cytoplasmic inheritance. As RRE measures the difference between the means of each pair of reciprocal crosses after an account for the average maternal difference between sire and dam lines/breeds has been taken (Henderson, 1948; Harvey, 1960; Eisen et al., 1983; Jakubec et al., 1988), the latter interpretation appears to be more relevant than the former one. Consistent with this, Fairful et al. (1983) defined RRE as a variation free of any general maternal and sex-linked effects. However, it might have complex interactions between maternal or sex-linked effects and autosomal effects (Eisen et al., 1967; Fairful et al., 1983).

According to Wearden et al. (1967), significant variation due to RRE for EN, HHEP, HDEP, and EM would suggest an interaction between sex chromosomes and autosomal chromosomes received from the other breeds involved in the cross. Comparably, Fairfull et al. (1983) reported significant RRE variation for HHEP, HDEP, and EW. Padhi (2010) also observed variations in reciprocal effects for AFE, EW, and duck-day egg production. However, these effects were non-significant for most egg production traits (Nestor et al., 2004; Razuki and AL-Shaheen, 2011; Soliman et al., 2020). For reciprocal crosses between FM and WL, Assefa et al. (2021) found reciprocal effects estimated at 3.50, − 3.50, and 1.00 for AFE, BWAFE, and EWAFE, respectively.

The present results generally highlight the importance of additive and non-additive genetic effects for egg production traits to be inherited. In most instances, KK and WL had a higher PE, with KK having the highest GCA. On the other hand, the ME was highest in FM and WL, where their cross (FM x WL) had a higher SCA and RRE than FM x KK. The KK x FM and FM x WL outperformed FM x KK and WL x FM, respectively, and their purebred contemporaries. It would be advantageous to mate females of FM x WL and KK x FM with any third breed to exploit heterosis in a three-way crossbreeding. It would also be possible to recommend a synthetic breeding program involving KK as a sire line and FM, WL, FM x WL, and KK x FM as a dam line.

## Data Availability

The data that support the findings of this study are included in the manuscript but are available from the corresponding author on reasonable request.
